# Supplementary material for: Olfactory bulb-cortex oscillations encode perceived odor intensity rather than concentration
Source: PLoS Biol. 2026 May 29;24(5):e3003810. doi: 10.1371/journal.pbio.3003810 (PMC13221048; doi:10.1371/journal.pbio.3003810)
Supplement: Attachment — Submitted filename: Norden_response_to_reviewers.docx [file pbio.3003810.s012.docx]

# Response to reviewers

Reviewer #1

The manuscript by Norden et al., uses EEG/EBG recordings to analyze neural activity from the olfactory bulb and piriform cortex during odor delivery in humans. From these experiments, the researchers conclude that 1) oscillatory dynamics in both the olfactory bulb and piriform cortex primarily encode subjective perceived intensity, rather than actual concentration, 2) gamma-band activity in the olfactory bulb encoding perceived intensity is transmitted to the piriform cortex, and 3) the piriform cortex sends top-down beta band feedback to modulate OB activity via phase-amplitude coupling.
The topic is of interest as the neural mechanisms underlying intensity coding in olfactory research is poorly understood. Research in animal models have primarily focused on concentration, rather than intensity coding, given the difficulty of obtaining perceptual measures of intensity in non-human subjects. In contrast, the current study correlates neural activity with perceived intensity on a trial-by-trial basis, providing unique insight into the mechanisms of intensity coding.

I just have one major concern that relates to finding #1.

**R1Q1.**      The authors find surprisingly little evidence of concentration-related modulation. This was somewhat shocking given the wealth of animal data supporting concentration-dependent coding in the olfactory bulb and piriform cortex. Thus, I am left wondering whether the authors can provide evidence that the vapor-phase concentration of these different liquid dilutions have 5-fold, 12-fold, and 14-fold differences between the low and high intensity stimuli. As the authors are no doubt aware, the relationship between liquid and vapor-phase concentrations are rarely linear at higher concentrations (Cometto-Muniz et al., 2003 and more). If these issues extend to the odorants/solvents used in this study, these positive deviations related to solvent-odorant interactions, could be skewing their data - thereby preventing an accurate test of concentration coding.

**Answer:** This is an important point regarding the relationship between liquid- and vapor-phase concentrations. We agree that nominal liquid dilutions do not necessarily translate linearly into vapor-phase concentrations, particularly at higher concentrations, as clearly exposed by Cometto-Muñiz et al. (2003). To address this question more directly, we have now measured the actual vapor-phase concentrations of all odorant stimuli used in the experiment using photon transfer reaction time of flight mass spectroscopy (PTR-TOF). These measurements confirmed that vapor-phase concentrations increased monotonically across the liquid dilution steps, but that the magnitude of the increase in vapor concentration differed across conditions and was not uniformly proportional to nominal liquid dilution factors, consistent with the nonlinear liquid–vapor relationships demonstrated by Cometto-Muñiz et al., 2003. Importantly, to directly answer the question posed by Reviewer #1, we then performed additional analysis using these empirically measured vapor-phase concentrations instead of liquid dilution values (Figure S7A-B). This analysis yielded results that were qualitatively and quantitatively indistinguishable from those reported in the manuscript based on calculated vapor-phase values. Thus, while the effective vapor-phase dynamic range was slightly reduced compared to the nominal liquid concentrations, explicitly accounting for this nonlinearity did not reveal concentration-dependent modulation in our neural measurements, suggesting that the absence of concentration coding effects in our data cannot be attributed to nonlinear liquid–vapor relationships. We have included these vapor-phase measurements and analyses in the supplementary materials and discuss them in the manuscript.

We added to the result section:

*“We also quantified absolute concentration values, measured at the olfactometer outlet (within the experiment, located about 2cm into the nose) using proton transfer reaction time of flight (PTR-TOF) mass spectroscopy. These analyses replicated the original finding: no cluster-corrected results of effective concentration on time–frequency amplitude (Supplementary Figure S7A-B).”*

We have added to the discussion:

*“However, this absence of concentration effects cannot be attributed to nonlinear liquid–vapor relationships* [*(Cometto-Muñiz et al. 2003)*](https://sciwheel.com/work/citation?ids=395046&pre=&suf=&sa=0&dbf=0)*. We directly measured vapor-phase concentrations using PTR-TOF mass spectrometry and repeated all analyses using empirically derived concentration values. These analyses yielded results indistinguishable from those based on nominal dilution levels.”*

And figure S7 can be found in the supplementary materials.

**Figure S7:** Power spectrum predicted by absolute concentration for A) OB and B) PC. Absolute concentration was measured with photon transfer reaction time of flight mass (PTR-TOF) spectroscopy at the outlet of the olfactometer. The values measured for the odors are: N-butanol: (low = 9 ppbV, high 12 ppbV), 5-nonanone (low=50 ppbV, high=300 ppbV), undecanal (low=7 ppbV, high=23 ppbV). Because nominal liquid dilutions do not necessarily translate linearly into vapor-phase concentrations, particularly at higher concentrations [(Cometto‑Muñiz et al., 2003)](https://sciwheel.com/work/citation?ids=395046&pre=&suf=&sa=0&dbf=0), the use of the vapor pressure metric can represent a limitation. Therefore, we measured the vapor-phase concentrations of all odorant stimuli used in the experiment deploying PTR-TOF mass spectrometry. These measurements confirmed that vapor-phase concentrations increased monotonically across the nominal dilution steps, but that the magnitude of the increase in vapor concentration differed across conditions and was not uniformly proportional to nominal liquid dilution factors, consistent with known nonlinear liquid–vapor relationships.

**R1Q1a.**      The authors state, "even when using an "effective concentration" metric (incorporating vapor pressure and dilution to linearize physical concentration across odorants), there was still no association between neural dynamics and concentration”. These analyses assume ideal vapor-phase concentration conditions, which are very unlikely to be accurate. Thus, this is not an accurate test of effective concentration coding. However, to be fair, many animal studies also incorrectly assume ideal vapor-phase concentrations. Although their tested concentration ranges are often larger, which may help mitigate this potential issue?

**Answer:** We agree that “effective concentration” metrics based on vapor pressure and nominal dilution assume idealized vapor-phase behavior, and that such assumptions are unlikely to be strictly valid under real stimulus delivery conditions. To directly address potential deviations from ideal vapor-phase behavior, as outlined above, we measured the vapor-phase concentrations of our set of odorants using PTR-TOF mass spectrometry. The measured values were then included in subsequent analyses (as previously mentioned) which returned the same results as the analysis included in the manuscript. These findings suggest that the absence of concentration-dependent modulation in our data cannot be attributed to inaccuracies in the effective concentration metric but rather reflects the neural response patterns observed across the empirically characterized vapor-phase concentration range tested here.

**R1Q1b.**      I attempted to look for vapor-/liquid-phase equations for these odorants and solvents. Jennings et al., 2023 did provide data for 1-butanol in diethyl phthalate. Their data indicate a ~6.3 fold rather than 5-fold change for this closely related odorant. (0.8% - 1342*0.8^1 = 1073 and 5% - 1342*5^1 = 6710). However, they also mentioned that this relationship only holds for concentrations below 3.5% (and not the 5% used in this study). I don't know if the authors can find any additional sources of information.

**Answer:** See R1Q1a.

**R1Q1c.**      A related concern stems from both the Jennings et al., 2023 and Commetto-Muniz et al., 2003 manuscripts. They observed that the relationship between liquid and vapor phase concentration can be "flatter" for longer chain odorants (ie exponents much lower than 1). If this is true for nonanone and undecanal, their vapor-phase concentration might be more "compressed" in comparison to the liquid concentration. This could theoretically skew the data and influence the statistical tests. However, this is just a supposition, I don't have any evidence for these particular odorants in diethyl phthalate.

**Answer:** See R1Q1a.


Reviewer #2

This study investigates how physical concentration vs. perceived intensity influence scalp electroencephalographic (EEG) activity in response to different odorants. Using advanced statistical approaches, the authors analyzed time-frequency power spectra in both the olfactory bulb (OB) and piriform cortex (PC). Their findings indicate that neural activity in these regions is driven primarily by perceived odor intensity rather than by physical concentration. More specifically, the authors report a sequence of gamma-band activity followed by beta-band activity within the first 2 seconds after odorant onset. They interpret this temporal pattern as reflecting an early bottom-up transmission of perceived intensity from OB to PC, followed by top-down modulation from PC back to OB via phase-amplitude coupling and transient beta bursts. This proposed dynamic is suggested to represent a neurocognitive mechanism underlying odor intensity coding. Overall, the manuscript is
well written and provides a sophisticated exploration of odor perception using human scalp EEG, contributing valuable findings to an area that remains relatively understudied. However, as outlined below, I have two major concerns regarding data analysis and interpretative overreach that should be addressed before I can recommend the paper for publication.

My first major concern relates to the analysis pipeline. More comprehensive analyses are needed to substantiate the reported results and to determine whether the findings truly capture the full picture. I outline below several specific points supporting this concern.

**R2Q1:**Lines 102-104, the authors state "Although the significant cluster began earlier in the OB, both regions showed intermittent beta-band peaks as early as ~200 ms after odor onset; these early peaks did not survive cluster correction." Then, on lines 112-115, they write "As in the beta band, early OB gamma effects (~200 ms; 70-80 Hz) were present but did not survive cluster correction. However, restricting analysis to an a priori window (100-500 ms) did yield a cluster-corrected effect, consistent with the hypothesis that early OB gamma carries information about perceived intensity." How was this a priori window defined? And why was this restricted window applied only to gamma-band activity related to perceived intensity in the OB? Visual inspection of Figure 2 suggests early activity within this window not only in the OB but also in the PC, and for both odorant concentration and perceived intensity in the latter, with no obvious differences between them. These early
responses may have been masked by the long-lasting clusters of late activity in the full 2-second cluster-based analysis. There is no clear rationale for restricting the analysis to an early window only for OB gamma activity associated with perceived intensity, particularly in light of the results from the later decoding analysis.

**Answer:**

We agree with your concern and have removed the analysis and the statement from the article. The text now reads:

*"As in the beta band, early OB gamma effects (~200 ms; 70-80 Hz) were present but did not survive cluster correction.”*

**R2Q2:** To ensure that the observed effects truly reflect neural processes specific to odor perception, the authors should include analyses from one or more control brain regions to demonstrate the absence of similar effects elsewhere. This would help confirm that the reported findings are not merely generalizable across non-target regions.

**Answer:**
We agree with the reviewer that control-region analyses are important to assess the anatomical specificity of the reported intensity–power effects. We therefore repeated the same intensity–power analyses in two control regions outside main olfactory circuitry: primary visual cortex (V1) and posterior cingulate cortex (PCC); one sensory-related processing area and one non-sensory area at a distance in-between these areas of interest as well as involved in attention and arousal processing. No significant intensity-related clusters were observed in either region under the same cluster-based permutation framework, supporting that the reported effects are not ubiquitous across cortex and are specific to the olfactory ROIs.

The following was added to the result section:

*“To ensure that these effects of perceived intensity on OB and PC power is not a cortex wide phenomenon, the same analysis was performed in two additional control regions: One sensory-related region (primary visual cortex, V1) and one higher order region involved in global arousal and attention (posterior cingulate cortex, PCC). No significant intensity-related clusters were observed in either region under the same cluster-based permutation framework (Supplementary Figure S8), supporting that the reported effects are not ubiquitous across cortex and are specific to the olfactory ROIs.”*

The following has been added to the discussion:

*“A critical question is whether effects show anatomical specificity. We therefore repeated the analyses in primary visual cortex and posterior cingulate cortex. No comparable intensity-related effects were observed under identical statistical criteria, arguing against a global anticipatory or task-structure account. Combined with the directionality and cross-frequency coupling observed within the OB–PC loop, these findings support the interpretation that late beta activity reflects recurrent refinement of perceived intensity rather than nonspecific expectancy effects.”*

The coordinates of the selected control regions have also been added to the methodology section:

*“Additional control regions were included in the analysis: primary visual cortex (V1; (x ±8, y-76, z+10), corresponding to Brodmann area 17 as defined in probabilistic cytoarchitectonic maps in MNI space (Amunts et al., 2000), and posterior cingulate cortex (PCC; (x±0, y−53, z+26), corresponding to BA23/31 based on established cytoarchitectonic mappings in MNI space (Vogt et al., 2006).”*

and Figure S8 has been added to the supplementary materials.

**Figure S8:** Power spectrum predicted by perceived odor intensity in primary visual cortex V1 (A) and posterior cingulate cortex PCC (B).

**R2Q3a:** The authors should make the raw data publicly available in a dedicated repository.

**Answer:** Thank you for pointing that out. Raw, anonymized EEG and MRI data are now available on OSF and can be reached via the existing link within the manuscript.

**R2Q3b:** In addition, more detailed data should be presented either in the main manuscript or the supplementary materials: Please provide the power spectra for each perceived intensity and each concentration rather than only statistical maps of the contrasts between conditions.

**Answer:**

We have added Figure S3 and S4 below to the supplementary materials and comment on the activation in the results section. The added text reads (with the whole paragraph added for context, the added text marked in yellow):

*“In contrast, perceived odor intensity revealed robust cluster-corrected effects in both neural regions. In the OB, beta-band (12–25 Hz) power was significantly related to intensity ratings from 600–1600 ms after odor onset (t = 3.51, p = .001, CI = [.0081, .0101]; Figure 2C). A similar beta-band effect in the PC occurred slightly later, from 850–2000 ms in the 15–30 Hz range (t = 3.03, p = .004, CI = [.0142, .0222]; Figure 2D). Although the significant cluster began earlier in the OB, both regions showed intermittent beta-band peaks as early as ~200 ms after odor onset; these early peaks did not survive cluster correction. Around 1 s into the trial, the association in the PC (t = 4.65, p = 3.36×10⁻⁶, CI = [.0757, .186]) was comparable to the ones in the OB (t = 4.08, p = 4.49×10⁻⁵, CI = [.0726, .210]). Activity in this region was also visible in the raw power spectrum, divided through a median split on perceived intensity (Supplementary Figure S4). Activation was also found in the raw power spectrum when dividing odor concentration into low and high (Supplementary Figure S3), but did, as noted above, not lead to any significant findings in the linear mixed-effects model.”*

and

*“Gamma-band results showed a similar pattern. In the OB, gamma power (70–100 Hz) was significantly related to perceived intensity between ~1100–1800 ms, and a later cluster emerged in the PC (80–100 Hz) from ~1400–2000 ms. Associations were comparable in the PC (t = 2.37, p = .022, CI = [.020, .064]) and the OB (t = 2.41, p = .02, CI = [.021, .061]). As in the beta band, early OB gamma effects (~200 ms; 70–80 Hz) were present but did not survive cluster correction. The activity was also here visible in the* *raw power spectrum, both for perceived intensity and concentration.”*

**Figure S3**: Power spectrum of low and high concentrations for OB and PC respectively.

**Figure S4**: Power spectrum of perceived intensity for OB and PC respectively.

**R2Q3c:** Figures S1 and S2 should be shown separately for the high and low concentrations to depict the relationship between concentration and perceived intensity.

**Answer:** We agree with the reviewers’ comment and have exchanged the previous Supplementary Figure S1 and Supplementary Figure S2 as seen below.

**Figure S1**: Histograms of participants’ low (A) and high (B) concentration odor intensity ratings. Solid line represents the density curve (kernel bandwidth=0.1).

**Figure S2**: Odor intensity ratings for each individual trial. Blue color indicates low concentration odors and red color indicates high concentration odors. Trials are sorted diagonally within each participant according to participant number (assigned in the order of entering the study).

My second major concern is that the authors make strong claims without sufficiently considering alternative interpretations or acknowledging the limitations of the evidence provided. I outline below several points that pertain to this concern.

**R2Q4:** Throughout the manuscript, the authors apply sophisticated statistical tools to localize EEG sources, infer the direction of information flow between the OB and PC, etc. However, these methods have intrinsic limitations to infer the neural mechanisms they intend to reveal (see e.g., Stokes & Purdon, 2017, PNAS, for the problems encountered in Granger causality analysis applied to neuroscience). Yet, the authors state lines 297-298 "These findings establish a mechanistic and temporally resolved model of how subjective olfactory intensity is constructed in the human brain". Relying solely on statistical modeling of scalp EEG to infer causal neurocognitive architecture is questionable, and this limitation should be clearly acknowledged.
**Answer:**

We agree that Granger causality and source-localized EEG do not provide definitive evidence of causal neural mechanisms, and that such inferences should be made cautiously (e.g., Stokes & Purdon, 2017). Our intention was not to claim proof of causal neurocognitive architecture, but rather to propose a temporally resolved model of OB–PC interactions that is consistent with the observed directionality of information flow and frequency-specific coupling.

To address this concern, we have revised the wording of our conclusions to avoid strong causal language (e.g., “establish”) and now explicitly acknowledge the inferential limitations of Granger causality and EEG source modeling in the Discussion. We emphasize that our findings provide evidence consistent with frequency-specific feedforward and feedback interactions during intensity perception, rather than definitive proof of underlying neural mechanisms. We believe this clarification appropriately constrains our claims while preserving the central contribution of the work.

The sentence now reads: “*Together, these findings support a temporally resolved model in which subjective odor intensity emerges through iterative feedforward and feedback interactions within the human OB–PC loop.”*

We have also added a paragraph in the discussion that highlights the importance of caution when interpreting the results (together with question R2Q5). It reads as follows:

*“Interpreting frequency-specific and directional interactions from non-invasive EEG signals requires care. Granger causality captures directed statistical dependencies between reconstructed signals rather than direct biological causation, and spectral peaks in Fourier-based analyses can reflect transient or non-sinusoidal events rather than sustained oscillations (Jasper 1948; Stokes and Purdon 2017; Doelling and Assaneo 2021). We therefore do not claim to have demonstrated causal neural mechanisms in a strict biological sense. What we do claim is that the pattern of effects, replicable across directed connectivity, phase–amplitude coupling, and single-trial burst dynamics, and consistent with established gamma and beta circuit mechanisms in animal models of olfaction (Brea et al. 2009; Osinski and Kay 2016; Dalal and Haddad 2022) is precisely what one would predict from an OB–PC circuit implementing feedforward and feedback processing of odor intensity. The convergence of independent measures, rather than any single analysis, constitutes the evidential basis for our interpretation. Together, these observations support the presence of temporally structured, frequency-specific interactions within the OB–PC circuit that are consistent with known anatomy and task timing.”*

**R2Q5:** In addition, several neurocognitive processes proposed by the authors to interpret the data are debated in the literature. For instance, the assumption that increases in power within a given frequency band reflect functional endogenous oscillatory activity remains disputed (e.g., Doelling & Assano, 2021, Plos Biol) and should be made with caution given the well-known "Fourier fallacy" (Jasper, 1948, Science). Similarly, the interpretation that high-frequency activity indexes local processing and low-frequency activity broader networks has been challenged by recent intracranial EEG findings (Jacques et al., 2022, eLife). Therefore, the manuscript would benefit from a more nuanced discussion of the neural interpretations.
**Answer:**

We agree that increases in spectral power do not necessarily imply sustained endogenous oscillations and may reflect non-periodic activity, as emphasized in discussions of the “Fourier fallacy.” However, our burst analysis finding narrow-band bursts several cycles long strongly hints at oscillatory activity. We also frame our frequency-specific interpretations within the context of olfactory system anatomy, timing, and converging evidence from animal and human studies, rather than as universal properties of neural oscillations. We have revised the Discussion to clarify that our interpretations do not rely on only on analysis of band-averaged power.

We also acknowledge that the commonly invoked association between frequency bands and spatial scales of processing (e.g., high-frequency local vs. low-frequency distributed) is a heuristic rather than a strict rule, and has been challenged by recent intracranial findings. We therefore frame our frequency-specific interpretations within the context of olfactory system anatomy, timing, and converging evidence from animal and human studies.

We have taken both points into consideration and added the two paragraphs to the discussion, where the first one is the same as for question R2Q4:

*“Interpreting frequency-specific and directional interactions from non-invasive EEG signals requires care. Granger causality captures directed statistical dependencies between reconstructed signals rather than direct biological causation, and spectral peaks in Fourier-based analyses can reflect transient or non-sinusoidal events rather than sustained oscillations (Jasper 1948; Stokes and Purdon 2017; Doelling and Assaneo 2021). We therefore do not claim to have demonstrated causal neural mechanisms in a strict biological sense. What we do claim is that the pattern of effects, replicable across directed connectivity, phase–amplitude coupling, and single-trial burst dynamics, and consistent with established gamma and beta circuit mechanisms in animal models of olfaction (Brea et al. 2009; Osinski and Kay 2016; Dalal and Haddad 2022) is precisely what one would predict from an OB–PC circuit implementing feedforward and feedback processing of odor intensity. The convergence of independent measures, rather than any single analysis, constitutes the evidential basis for our interpretation. Together, these observations support the presence of temporally structured, frequency-specific interactions within the OB–PC circuit that are consistent with known anatomy and task timing.”*

And:

*“Importantly, the association between gamma and feedforward processing, and beta and feedback processing, should be regarded as a functional heuristic rather than a strict rule. We therefore base our interpretation not on frequency alone, but on the combined evidence of temporal ordering, directionality, behavioral relevance, and consistency with known olfactory circuitry. Within these constraints, the OB–PC dynamics observed here provide converging evidence for recurrent feedforward and feedback interactions underlying subjective odor intensity processing, paralleling communication principles described in other sensory systems (Bastos et al. 2015).”*

**R2Q6:** One clear example of an alternative interpretation relates to the claim on lines 250-251: "revealing that perception is manifested remarkably early in the human olfactory hierarchy". The temporal dynamics reported in the study, within a 2-s window, are in fact relatively slow compared to what is known about the speed of sensory perception in general, and olfactory perception in particular. In rodents, odor perception can occur within approximately 500 ms (e.g., Bolding & Franks, 2017, eLife). Although human processing may be slower due to larger brain size, behavioral latency studies suggest that 1 s is sufficient for many olfactory processes (reviewed in Olofsson, 2014, Front Psychol), and this same review notes that "major olfactory decisions may be confined to a rapidly unfolding cascade of inter-connected processing steps within a 500 ms interval" (p. 6).

Accordingly, what exactly does the late beta activity observed from 1 s onward represent? Its latency suggests that it may be more related to the task structure, namely waiting for 2 seconds before providing an intensity judgment, rather than odor perception per se. Classic work by Walter (1964, Nature) showed that sensorimotor associations and expectancy elicit a slow negative potential, the contingent negative variation (CNV), and later studies have linked CNV amplitude and latency to beta‑band power in time-frequency analyses (e.g., Bickel et al., 2012, NeuroImage). Thus, the late beta activity reported here may disappear under passive sniffing conditions without an upcoming explicit task. As noted above, including analyses of a control brain region not expected to show odor‑related responses would help clarify this issue, and this possibility should be discussed.
**Answer:**

We agree that the phrase “remarkably early” may have been interpreted as referring to temporal latency. Our intention was instead to refer to the anatomical stage of processing — namely, that perceptual representations are detectable already at the level of the olfactory bulb, traditionally considered a predominantly sensory relay. We have revised the wording to clarify that “early” refers to hierarchical position rather than speed of processing.

It now reads: *“These findings challenge the traditional view that early olfactory processing reflects stimulus concentration and instead reveal that perception is already manifested at the earliest hierarchical stages of the human olfactory system.”*

Regarding the late beta activity (~1 s onward), we acknowledge that its latency extends beyond the time window typically associated with initial odor detection and could overlap with task-related processes such as expectancy or preparation for the intensity judgment. We therefore agree that the late beta activity likely reflects post-initial processing rather than primary sensory encoding.

However, several observations argue against a purely task-general explanation (e.g., CNV). First, new control-region analyses in V1 and PCC (as suggested by the reviewer, see R2Q2), using the identical cluster-based permutation framework, did not reveal comparable late beta effects, suggesting that the phenomenon is not a widespread anticipatory signal. Second, the beta activity was embedded within a frequency-specific OB–PC interaction pattern, including direction-specific connectivity and phase–amplitude coupling, which is difficult to reconcile with a global expectancy account.

We therefore interpret the late beta dynamics as reflecting recurrent or evaluative processing of perceived intensity within the olfactory circuit, while acknowledging that future studies employing passive paradigms will be valuable for further dissociating perceptual and task-related contributions.

The following paragraph has been added to the Discussion (same as for R2Q2):

*“A critical question is whether effects show anatomical specificity. We therefore repeated the analyses in primary visual cortex and posterior cingulate cortex. No comparable intensity-related effects were observed under identical statistical criteria, arguing against a global anticipatory or task-structure account. Combined with the directionality and cross-frequency coupling observed within the OB–PC loop, these findings support the interpretation that late beta activity reflects recurrent refinement of perceived intensity rather than nonspecific expectancy effects.”*

Minor points
**R2Q7:** I do not understand whether "perceived intensity" is treated as a continuous variable or as a dichotomized variable (i.e., median-split) in the first analysis using a linear mixed-effects model. This should be made explicit. If the authors indeed used a median split, they should additionally provide an analysis treating perceived intensity as a continuous predictor to verify that the results hold, given the well-documented statistical drawbacks of median-split procedures (e.g., MacCallum et al., 2002, Psych Methods).
**Answer:**

We do agree that median-split procedures have well-documented statistical limitations. Accordingly, perceived intensity was treated as a continuous variable in all analyses, including the linear mixed-effects models. The only exception was the analysis of the coherence spectrum, and now per reviewer request, Figure S4. Because coherence estimates are inherently based on averaging across trials, we were forced to employ a median split in this specific analysis to enable a comparison between relatively higher- and lower-intensity perceptions. Importantly, all inferential analyses central to our conclusions model perceived intensity as a continuous predictor, ensuring that the reported effects do not depend on dichotomization.

*“The spectral power was used as the outcome variable while perceived intensity or concentration, together with perceived valence, sniff amplitude and area under the curve (AUC) of the sniff signal, were used as fixed effects and perceived intensity and valence were treated as continuous variables.”*

We also clarified it in the result section:

*“In contrast, perceived odor intensity revealed robust cluster-corrected effects in both neural regions when continuous intensity ratings were modeled as a fixed-effect*.”

**R2Q8:** Regarding the interpretation of differences between OB and PC, the authors write on lines 104-107: "Around 1 s into the trial, the association was stronger in the PC (t = 4.65, p = 3.36×10⁻⁶, CI = [.0757, .186]) than in the OB (t = 4.08, p = 4.49×10⁻⁵, CI = [.0726, .210]), suggesting that the PC holds a richer representation of perceived intensity at this time point."  Similarly, on lines 110-112, they state: "Associations were comparable in magnitude for PC (t = 2.37, p = .022, CI = [.020, .064]) and OB (t = 2.41, p = .02, CI = [.021, .061])." However, these statements appear to rely solely on descriptive differences in t-values. To substantiate claims about differential sensitivity between OB and PC, the authors should directly compare power spectra between regions.

**Answer:**

We agree with the reviewer that differences in t-values across separate models are descriptive and do not constitute a formal test of regional differences. To directly test whether perceived-intensity sensitivity differs between OB and PC, we fitted a single linear mixed-effects model including both regions and an explicit Region × Intensity interaction term. This interaction provides a direct statistical comparison of the intensity–power association (i.e., slope) between OB and PC. It did not show any statistical differences between the regions, and we have hence removed those claims from the result section.

The text now reads:

*“Around 1 s into the trial, the association in the PC (t = 4.65, p = 3.36×10⁻⁶, CI = [.0757, .186]) was comparable to the ones in the OB (t = 4.08, p = 4.49×10⁻⁵, CI = [.0726, .210]).”*

And:

*“Associations were comparable in the PC (t = 2.37, p = .022, CI = [.020, .064]) and the OB (t = 2.41, p = .02, CI = [.021, .061]).”*
