# Supplementary material for: Olfactory bulb-cortex oscillations encode perceived odor intensity rather than concentration
Source: PLoS Biol. 2026 May 29;24(5):e3003810. doi: 10.1371/journal.pbio.3003810 (PMC13221048; doi:10.1371/journal.pbio.3003810)
Supplement: Attachment — Submitted filename: Response to reviewers_R2.docx [file pbio.3003810.s013.docx]

Response to reviewers

# Reviewer 2:

**R2Q1:** In the previous review, I emphasized a lack of justification for restricting the cluster-based analysis to an early time window only for OB gamma activity related to perceived intensity. In response, the authors simply removed the analysis. However, as I already noted, Figure 2 shows early neural activity within the early 100-500 ms window in the beta band not only in the OB but also in the PC, and for both odorant concentration and perceived intensity in the PC. I also pointed out that early effects may be obscured by the presence of late and long-lasting significant clusters. Hence, my comment was rather to encourage the authors to examine early activity across all conditions and all frequency bands (see also my final comment below).

**Answer:** We thank the reviewer for this clarification. We understood the previous comment as a implicit critique towards restricting our analysis to gain unjustified power and therefore removed them. However, following the latest suggestion, we reanalyzed the data using a restricted time window (0–500 ms) across all frequency bands and conditions. This analysis did not reveal any cluster-corrected effects for either perceived intensity or odor concentration in the OB or PC. The reporting of the effects in this restricted time window in the original proposed article was hence, unfortunately, erroneous. Our previous cluster correction implementation was unfortunately using too lenient parameters. We have now corrected throughout, but this is the only noticeable change of results due to the restricted analyses space. That said, although some early fluctuations are visible in the mixed-effects time-frequency plots, these did not reach significance when analyzed independently, suggesting that they reflect subthreshold or activity with high variability rather than robust, consistent effects across participants. To ensure the integrity of the results, we reanalyzed the main findings over the full time window, which were reproduced in full.

However, the comment from Reviewer 1 is a valid and interesting comment that we think should be addressed in the manuscript. Therefore, we have now included this in the Results section where we write:

*“To account for the rapid dynamics of olfactory processing, we repeated the analyses using a restricted time window of the first 500 ms following odor onset. This analysis did not reveal any significant cluster-corrected effects for either perceived intensity or odor concentration.”*

**R2Q2:** I appreciate the newly provided supplementary figures showing the raw power spectra for odor concentration and perceived intensity in both the OB and PC. Surprisingly, these figures suggest a strong effect of odor concentration on late (1-2 s) gamma activity, particularly in the OB, while the effect of perceived intensity does not visually appear markedly different from that of concentration. While visual inspection alone is not sufficient to draw conclusions, it is nonetheless puzzling that the pronounced dissociation between the effects of odor concentration and perceived intensity reported in the statistical analysis is not more clearly reflected in the raw data. Could the authors elaborate on this discrepancy?

**Answer:** We agree that a visual inspection of the raw power spectra can give the impression of similar effects for odor concentration and perceived intensity, particularly in the late gamma range. However, this type of visual inspection of average spectra does not account for variability across trials and participants, nor for the multiple-comparison correction applied in the statistical analysis. This apparent discrepancy arises because the dissociation between perceived intensity and concentration is driven by differences in consistency and statistical robustness across participants, rather than by large differences in the average spectral magnitude.

However, given the visual appearance and obvious interest from an informed reader, to examine this more directly, we performed an additional cluster-based permutation test on the concentration contrast within the same time–frequency window. This new analysis revealed a small area of late beta-band cluster (~0.8–1.2 s) that reached cluster-level significance (t = 3.5, p = .049, CI = [.0094, .0176]), partially overlapping with the time window in which intensity-related effects were observed. Given the natural correlation between odor concentration and perceived intensity, paired with the temporally late time window, this is not a surprise. The late timing of this effect suggests that it may reflect processes that are not purely sensory (bottom-up mediated by receptor activity differences due to concentration) and likely involve perceptual or task-related components, i.e. more cognitively mediated.

Critically, this concentration-related effect was not supported by the linear mixed-effects model, which did not reveal any significant association between concentration and time–frequency power. In contrast, perceived intensity showed consistent and converging effects across multiple analytical approaches, including the linear mixed-effects model, cluster-based permutation testing, and additional measures such as phase–amplitude coupling and burst dynamics. We also note that median-split analyses of perceived intensity showed similar spectral patterns, but these did not survive cluster correction, likely reflecting reduced sensitivity due to dichotomization of a continuous variable.

Taken together, these results suggest that while some late concentration-related activity may be present, it is less consistent and less robust across analytical approaches than the effects of perceived intensity. We have clarified this point in the manuscript. Our main conclusion, that oscillatory dynamics in the OB and PC primarily reflect perceived intensity rather than physical concentration, remains supported by the overall pattern of results.

We have added this text to the result section of the article:

*“These effects were also visible in the raw power spectra (Supplementary Figure 3, Supplementary Figure 4). When trials were divided by perceived intensity using a median split, one significant cluster was observed in the PC in the gamma band (~30 Hz, 1500–1800 ms; t = 3.7, p = .018, CI = [.0064, .0116]). Similarly, dividing trials by low versus high odor concentration revealed a significant cluster in the beta band (~25 Hz, 800–1200 ms; t = 3.5, p = .049, CI = [.0094, .0176]) overlapping with the time window identified in the mixed-effects model for perceived intensity.”*

And this text to the discussion:

*“Notably, an additional cluster-based analysis revealed a small late concentration-related effect in the beta range that overlapped temporally with the intensity-related activity. However, this effect was not observed in the linear mixed-effects model and was less consistent across analytical approaches. The temporal overlap with intensity-related dynamics suggests that such activity may reflect shared or interacting processes rather than a distinct encoding of physical concentration. Thus, while some late concentration-related signatures may be present, they appear weaker and less robust than the effects associated with perceived intensity.”*

**R2Q3:** Previously, I proposed that the late beta activity could be related to task structure rather than odor perception per se because the requirement to wait for 2 seconds before reporting an intensity judgment may elicit expectancy processes measurable in beta-band activity. I therefore suggested that the late effects described in the manuscript might not appear in a passive sniffing paradigm. The authors acknowledged in their reply that passive tasks could be valuable but did not mention it in the manuscript. Rather, based on additional analyses showing that the effects are confined to the olfactory system, they have added a short rebuttal arguing that this specificity rules out a global, unspecific anticipatory mechanism.

While I agree that the new analyses effectively demonstrate that the effects are specific to the olfactory regions, they do not dismiss the involvement of expectancy-related processes. These processes are not necessarily unspecific. Previous studies, including the study I previously cited (Bickel et al., 2012, NeuroImage), show that late expectancy-related neural activity can covary with stimulus content when the content is relevant for the upcoming behavioral decision. This is directly analogous to the present study, in which perceived intensity determines the behavioral response. Therefore, the argument that expectancy processes would necessarily be global or unspecific is not convincing.

In addition, as I previously mentioned, and as reviewed by Olofsson (2014, Front Psychol), odor processing proceeds rapidly, with high-level perceptual and semantic components unfolding well within the first second following odor onset. Because this time course is based on behavioral reaction times, which include motor components, the underlying neural processes are likely to occur even earlier than 1 second. These points reinforce the importance of examining the early window more precisely (see my first comment) and the manuscript should explicitly discuss this limitation, acknowledge that late activity may conflate perceptual and task-related components, and note that passive paradigms would be beneficial in future studies.

**Answer:** We agree that expectancy-related processes need not be global or unspecific and may indeed covary with task-relevant stimulus dimensions such as perceived intensity. Accordingly, we cannot exclude the possibility that the late beta activity reflects, at least in part, task-related processes associated with the upcoming intensity judgment.

At the same time, we note that the observed beta activity is embedded within a broader pattern of OB–PC interactions, including frequency-specific directionality and phase–amplitude coupling, as well as temporally aligned burst dynamics, all of which are systematically related to perceived intensity. These features suggest that the late beta activity is not an isolated phenomenon but part of a structured network interaction within the olfactory system. We do also note that animal studies have seen similar beta activity related to odor concentration at the similar time points as we in a passive paradigm. (Lam et al., 2000, J Neurosci)

Importantly, our control-region analyses demonstrate that these effects are not ubiquitous across cortex, arguing against a purely global anticipatory account. However, as the reviewer correctly notes, this does not exclude the possibility of content-specific expectancy effects.

In line with this, we have revised the Discussion to explicitly acknowledge that late activity may reflect a combination of perceptual and task-related processes, and that passive paradigms without an explicit behavioral report would be a future valuable contribution to disentangle these components. We also note that the absence of significant effects in the early (0–500 ms) window further limits our ability to isolate purely perceptual processing in the present design.

We have added the following to the discussion:

*“However, this does not exclude the involvement of content-specific expectancy mechanisms. Accordingly, the late beta activity may reflect an interaction between perceptual refinement and task-related processes within the olfactory system. Future studies using passive odor presentation or delayed-response paradigms will be important to disentangle these components.”*
